# Supplementary figures and images for: Chromosome-level genome assemblies for two quinoa inbred lines from northern and southern highlands of Altiplano where quinoa originated
Source: Front Plant Sci. 2024 Aug 19;15:1434388. doi: 10.3389/fpls.2024.1434388 (PMC11366598; doi:10.3389/fpls.2024.1434388)

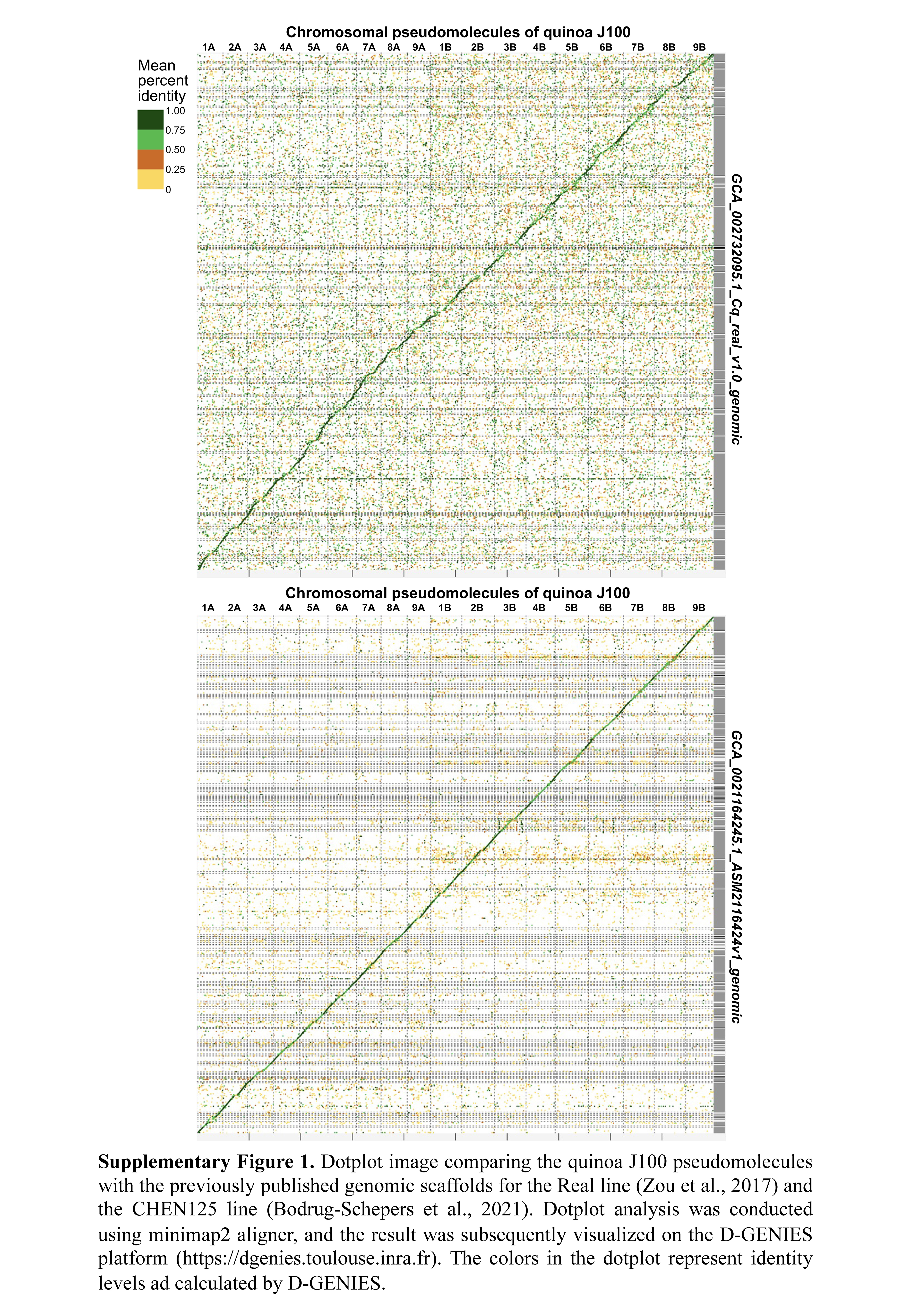

Supplement: Supplementary file 2 [file Image1.jpeg]

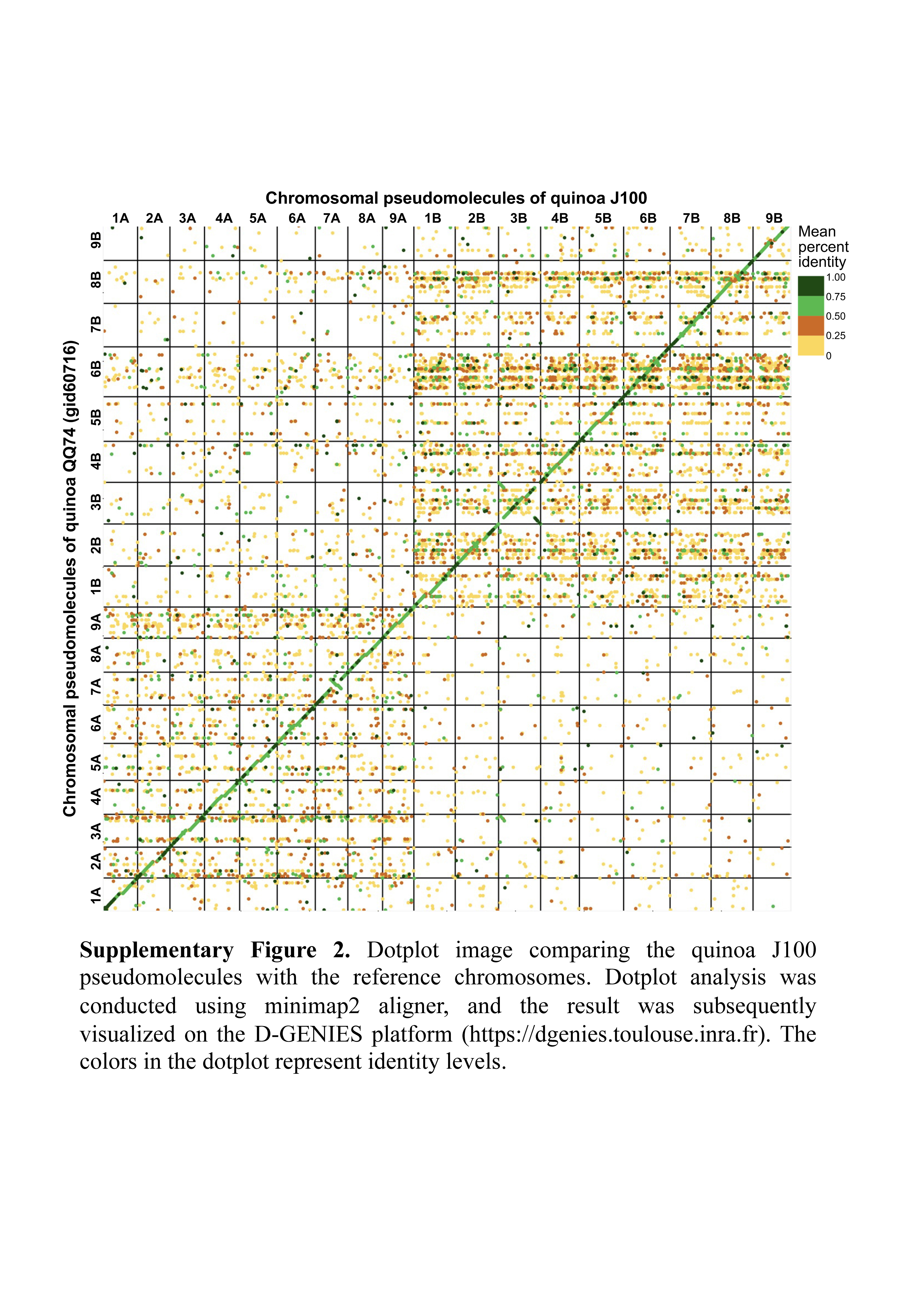

Supplement: Supplementary file 3 [file Image2.jpeg]

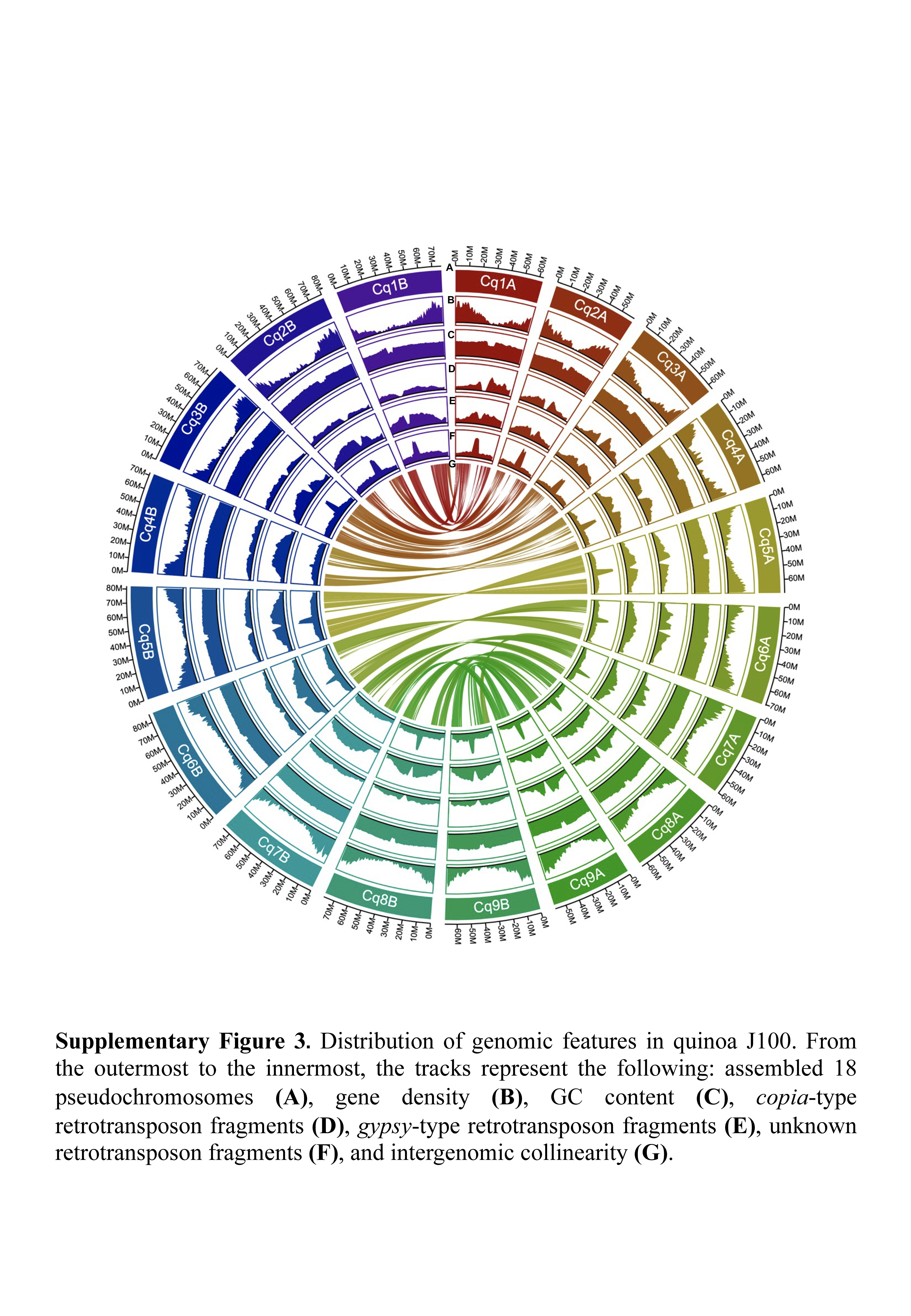

Supplement: Supplementary file 4 [file Image3.jpg]

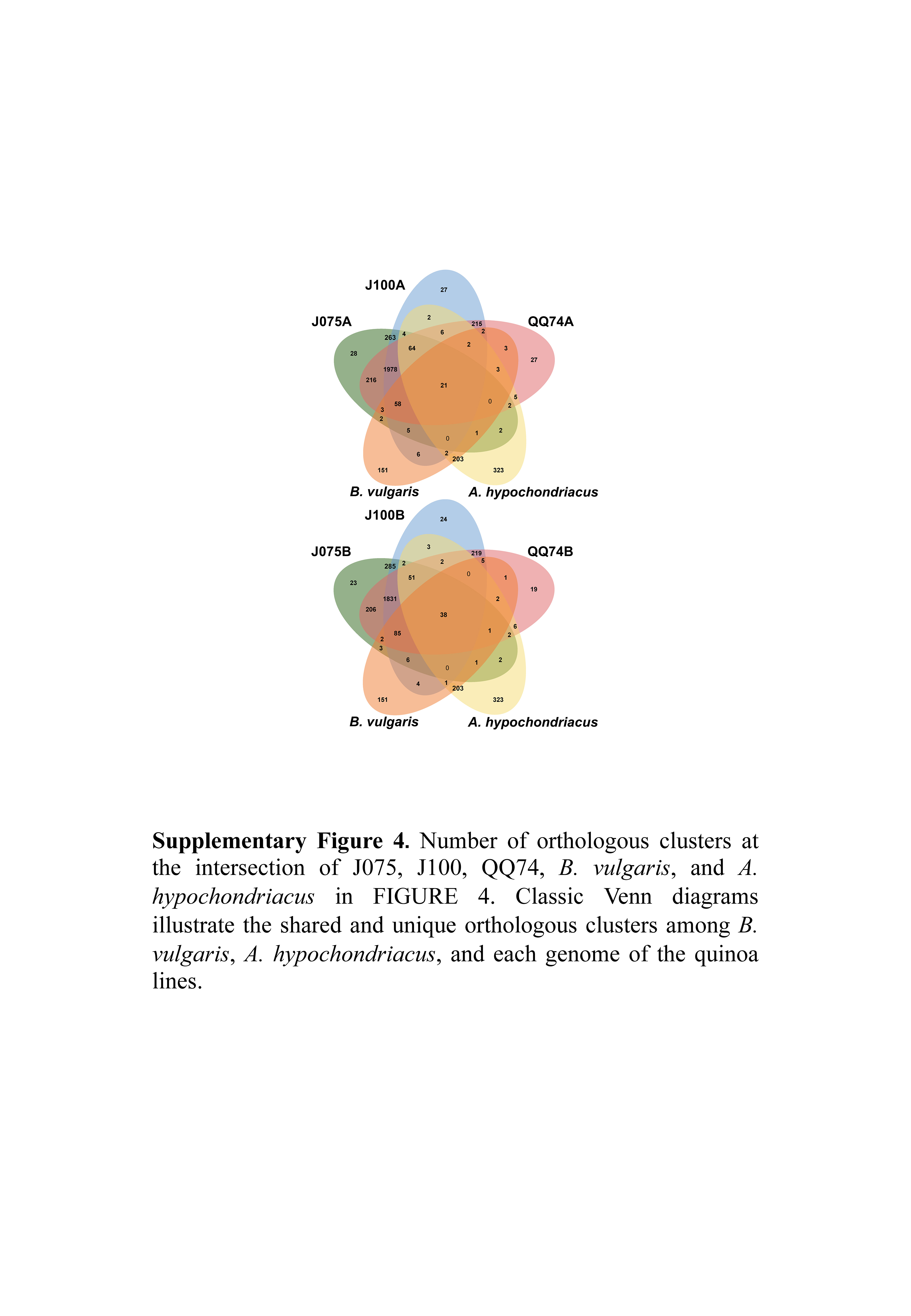

Supplement: Supplementary file 5 [file Image4.jpeg]
